# Development and validation of the physician self-efficacy to manage emotional challenges Scale (PSMEC)

**DOI:** 10.1186/s12909-024-05220-9

**Published:** 2024-03-04

**Authors:** Maria Weurlander, Linda Wänström, Astrid Seeberger, Annalena Lönn, Linda Barman, Håkan Hult, Robert Thornberg, Annika Wernerson

**Affiliations:** 1 Department of Education, Stockholm University, Stockholm, Sweden; 2 Department of Computer and Information Science, Linköping University, Linköping, Sweden; 3 Department of Clinical Science, Intervention and Technology, Karolinska Institutet, Stockholm, Sweden; 4 Department of Learning in Engineering Sciences, School of Industrial Engineering and Management, KTH Royal Institute of Technology, Stockholm, Sweden; 5 Department of Behavioural Sciences and Learning, Linköping University, Linköping, Sweden

**Keywords:** Physician self-efficacy, Medical students, Emotional challenges, Self-efficacy questionnaire, Validation, Measurement invariance

## Abstract

**Background:**

Medical students experience emotional challenges during their undergraduate education, often related to work-based learning. Consequently, they may experience feelings of uncertainty and self-doubt, which can negatively affect their well-being. Therefore, it is crucial to support students’ development of their ability to manage distressful situations. Self-efficacy beliefs may be a central aspect of supporting them in this development, and have been shown to relate to resilient factors such as students’ motivation, learning, and well-being.

**Methods:**

We constructed a scale to measure medical students’ physician self-efficacy to manage emotional challenges during work-based learning, the PSMEC scale. The aim of the present study was to evaluate some of the psychometric properties of the PSMEC scale. The scale consists of 17 items covering five subscales: (1) medical knowledge and competence, (2) communication with difficult patients and delivering bad news, (3) being questioned and challenged, (4) educative competence in patient encounters, and (5) ability to establish and maintain relationships with healthcare professionals. Data were collected from 655 medical students from all seven medical schools in Sweden. To investigate the scale’s dimensionality and measurement invariance with regard to gender and time in education, single and multiple group confirmatory factor models were estimated using techniques suitable for ordered categorical data. Measures of Cronbach’s alpha were calculated to evaluate internal consistency.

**Results:**

The scale showed good internal consistency on both the global dimension and the five subdimensions of self-efficacy. In addition, the scale was shown to be measurement invariant across genders and times in education, indicating that the scale means of male and female medical students and the scale means of students at the middle and end of their education can be compared.

**Conclusions:**

The physician self-efficacy to manage emotional challenges scale demonstrated satisfactory psychometric properties, with regards to dimensionality, internal consistency, and measurement invariance relating to gender and time in education, and this study supports the usefulness of this scale when measuring self-efficacy in relation to emotional challenges.

**Supplementary Information:**

The online version contains supplementary material available at 10.1186/s12909-024-05220-9.

## Introduction

Medical students experience several challenges during medical education, especially during work-based learning [1]. Many of these challenges generate emotions [2–6] and concern meeting severely ill and suffering patients and being uncertain of one’s own medical knowledge and skills. Medical students may feel doubt about whether or not they have sufficient knowledge and skills, and they express fear of making medical mistakes [4, 7, 8]. Patient encounters can also be challenging for other reasons. The doctor‒patient relationship builds on trust and good communication, which in turn affects patient satisfaction and health outcomes [9]. However, patient communication may be challenging [10]. For example, medical students sometimes experience stress and insecurity when delivering bad news to severely ill patients [11, 12] or caring for ‘difficult patients’, i.e., patients who are uncooperative or uninterested [13, 14]. Furthermore, in their professional role as physicians, they may sometimes be challenged in their medical decisions or advice by patients, relatives, or other healthcare professionals, which may undermine trust and communication in the doctor‒patient relationship [15, 16]. Another aspect of doctor‒patient communication is that physicians are expected to explain and help patients understand their diseases and motivate them to follow medical advice and treatment plans [9, 17]. The challenges students face arise not only in relation to patient encounters. Students may also witness what they perceive as unprofessional behaviors among healthcare professionals or experience a detached medical culture [3, 4, 18–20]. Relationships with colleagues and other healthcare professionals are important, especially because medical practice inevitably involves teamwork [21]. Observing unprofessional behavior among healthcare professionals may therefore be problematic for students. Furthermore, the clinical placements are often short, leaving little time to establish trusting relationships with supervisors and other healthcare professionals, which may be challenging for the students who sometimes feel excluded from the healthcare team [4, 22].

Dealing with stress and other negative emotions is important not only for students’ well-being but also for their learning [23–25]. Emotional challenges may result in self-doubt, shame and imposter syndrome, which in turn are associated with several components present in burnout [7, 23, 26, 27]. Students who doubt their own medical knowledge and clinical competence may experience feelings of inadequacy and question that they have what it takes to become a skilled physician [4, 23, 24]. Therefore, identifying factors associated with self-doubt and imposter syndrome, such as lack of self-efficacy beliefs, is important and can provide information useful for medical schools to address the strengthening of students’ professional identity.

In recent years, medical students’ self-efficacy beliefs have gained increased interest from researchers [28], and there is a growing body of research demonstrating the importance of self-efficacy for medical students’ motivation, learning, and achievement [29–34]. Students’ self-efficacy concerns the beliefs or confidence in their capability to perform a certain task, take adequate action or behave in a way that results in a desirable outcome [35, 36]. Self-efficacy has also been described as central to internally induced medical decision-making [37]. Furthermore, students’ self-efficacy beliefs relate to their well-being and resilience. Self-efficacy beliefs have been found to negatively correlate with stress, anxiety and depressive symptoms [38, 39]. In addition, feelings of inadequacy may influence achievement negatively, and ‘skills can be easily overruled by self-doubts, so that even highly talented individuals make poor use of their capabilities under circumstances that undermine their beliefs in themselves’ [36, p.37]. From the research above, self-efficacy emerges as essential to medical students’ learning and professional development. This is particularly interesting because research points to the common experience of emotional challenges, feelings of inadequacy and medical students’ and junior doctors’ doubts about their capabilities. To further explore medical students’ beliefs in themselves in distressful situations, we developed a scale measuring self-efficacy beliefs related to the emotionally challenging situations students reported in previous studies (i.e. [3, 4, 27]). In this paper, we present some psychometric properties of the Physician Self-efficacy to Manage Emotional Challenges Scale (PSMEC).

### Measuring self-efficacy

Self-efficacy, according to Bandura’s definition [35, 36], refers to beliefs about one’s *capabilities* and not expectations of outcomes. Furthermore, self-efficacy is future-oriented and context dependent, meaning that self-efficacy beliefs concern a person’s belief in the capability to perform a task, act or behave in a successful and goal-directed way in a particular context. However, in a recent review, Klassen and Klassen [28] conclude that many studies exploring self-efficacy are conceptually or operationally flawed.

There are to date various inventories measuring self-efficacy, some measuring general self-efficacy independent of context [40], and others tailored to specific contexts or tasks [41–43]. The OCCupational Self-EFFicacy scale (OCCSEFF) [44] focuses on work-related self-efficacy on a medium-level generality, enabling comparisons between professions. Regarding self-efficacy in the medical context, two inventories have been developed from comprehensive frameworks describing medical competencies, such as CanMEDs and ACGME core competencies: the Medical Achievement Self-efficacy Scale (MASS) [41] and Self-efficacy in Medical School [42]. The MASS inventory [41] is based on the competencies defined in CanMEDs [45] and captures the medical students’ self-efficacy beliefs of all medical education dimensions: medical expert, communicator, collaborator, manager, health advocate, scholar, and professional. Similarly, the Self-efficacy in Medical School inventory [42] was developed based on the ACGME core competencies [46]: patient care, medical and population health knowledge, interpersonal and communication skills, practice-based learning and improvement, professionalism, and systems-based practice. Exploratory factor analyses revealed three factors: patient care, interpersonal skills, and evidence-based medicine [42]. The aforementioned instruments do not explicitly focus on students’ emotional challenges. Therefore, and in contrast to previously existing instruments, we developed a scale specifically addressing medical students’ experienced emotional challenges. This scale thus adds important knowledge to medical students’ challenges considering that emotional distress may cause feelings of inadequacy and self-doubt about whether they have what it takes to become a skilled physician. The aim of the present study is to describe this instrument, the Physician Self-efficacy to Manage Emotional Challenges scale (PSMEC), and to evaluate some of its psychometric properties, namely its dimensionality, internal consistency, and measurement invariance with regards to gender and time in education.

## Methods

### Development of the scale

Based on Bandura’s theory of self-efficacy [36], we constructed a questionnaire concerning medical students’ physician self-efficacy to manage emotional challenges in work-based education. The items were developed using interview data from medical students regarding emotional challenges [3, 4] and were informed by the literature regarding medical students’ challenges and other instruments measuring professional self-efficacy (i.e., teacher self-efficacy [47] and occupational self-efficacy scale [44]). Congruent with Bandura’s theory, we aimed to develop an instrument that is domain specific, i.e., focusing on the domains previously identified as challenging for medical students and that may contribute to their feelings of inadequacy and self-doubt. The five domains regarding communication, being questioned, medical and educative competence, and ability to establish and maintain relationships with healthcare professionals are presented below.

*Communication with difficult patients and delivering bad news*– This dimension refers to communication with difficult patients and delivering bad news. Difficult patients may be defined as patients who are uncooperative, upset or uninterested.

*Being questioned and challenged*– This dimension concerns being questioned or challenged in medical decisions or advice by patients, relatives, or other healthcare professionals.

*Medical knowledge and competence*– This dimension refers to having sufficient medical knowledge and skills.

*Educative competence in patient encounters*– This dimension refers to the ability to explain and help patients understand their diseases and motivate them to follow medical advice and treatment plans.

*Ability to establish and maintain relationships with healthcare professionals*– This dimension concerns the capability to maintain good working relationships with colleagues and other healthcare professionals.

The domains above correspond to several dimensions in frameworks such as CanMEDs [45] and ACGME [46]; however, the items in our PSMEC scale focus on situations in the clinical context that students may find emotionally challenging [3, 4], as described previously. Our scale included the following 17 items (see Table 1) covering self-efficacy to manage situations captured by the five domains described above.

The response scale for each item was on a six-point Likert-type scale from 1 = strongly disagree to 6 = strongly agree. The items were constructed in Swedish, and the validity analysis presented here was conducted on the Swedish version of the scale. Physicians and experienced medical educators in the research team (AW, AS and ALL) assured the content validity of each item. We evaluated the properties of both the global level of the physician self-efficacy scale and of each subscale. This was to investigate the robustness of the scale on both the global and subscale levels and to ensure that both the global and the subconstruct of students’ physician self-efficacy can be measured by the scale. A global measure of self-efficacy can be useful for investigating self-efficacy in relation to other constructs, whereas measuring self-efficacy on a subscale level provides a more nuanced picture and by some argued to increase the accuracy of prediction regarding student outcomes [36, 48]. The psychometric properties of the scale are described in the results section.

**Table 1:** Subscales and items of the physician self-efficacy to manage emotional challenges scale (PSMEC)

Subscale	Items
Patient communication	3. I can communicate with difficult patients 5. I can handle difficult questions from patients
	9. I am confident in my ability to meet a patient/relative who expresses strong emotions
	13. I am good at calming patients down 14. I am good at delivering bad news to the patient
	17. I am good at forming good relations to patients
Being questioned	7. I can handle being questioned by patients 10. I can handle being questioned by healthcare professionals
Medical competence	1. I feel confident that I have sufficient knowledge
	4. I am good at the practical skills that are necessary in my role as a physician
	8. I feel confident in how to handle my knowledge gaps 12. I believe in my ability to make sound medical decisions
Educative competence	2. I can make patients follow advice and recommendations
	11. I am good at judging whether the patient has understood my information
	15. I can explain in a way that patients understand
Relationships w healthcare professionals	6. I am able to contribute to a positive collaboration in the healthcare team
	16. I am good at establishing good relations to the healthcare professionals

### Data collection and sampling

We aimed to collect data from students with different levels of work-based education experience. Therefore, we invited students from all seven universities offering medical education in Sweden to participate in the study. Furthermore, we included students from two different cohorts: in the middle of their undergraduate studies and at the end of their five- and a-half-year undergraduate studies. The students who were halfway through their undergraduate studies had recent experiences of entering work-based education. Students who were at the end of their studies, on the other hand, were sampled because they had more experience from participating in clinical work. Data were collected before the COVID-19 pandemic. A web-based version of the questionnaire was first distributed through e-mail to the medical students. Because the response rate was low (*n* = 463, 20% response rate) even after three e-mail reminders, a paper version was sent to students who had not responded to the web-based questionnaire. The low response rate may have been due to students’ reluctance to open links in e-mails, low interest in the survey content or the structure of the survey [49]. A total of 2,286 medical students received the questionnaire (web version, and for some, also paper version), and 655 students provided complete responses to the 17 items (28% response rate). A majority were women (*n* = 397, 61%), which reflected the overall gender distribution of medical students at a national level [50]. A total of 335 (51%) of the students were in the middle of their studies, and 320 (49%) were studying their final year of the undergraduate medical program. Students from all seven medical schools answered the questionnaire.

### Psychometric evaluation procedure

As described in the Methods section, the scale was designed to measure five dimensions of medical students’ physician self-efficacy to manage emotional challenges: (1) medical knowledge and competence, (2) communication with difficult patients and delivering bad news, (3) being questioned and challenged, (4) educative competence in patient encounters, and (5) ability to establish and maintain relationships with healthcare professionals. The dimensionality of the scale was evaluated using confirmatory factor analysis (CFA) [51] using the lavaan package, version 0.6.9.1633 [52] in R, version 4.0.2 [53]. A hierarchical CFA model with five first-order factors (corresponding to the five domains) and one second-order global factor was estimated using the cat-ULS estimator, which is suitable for ordered categorical data [54–56]. The cat-ULS estimator was used for all other CFA analyses as well. Because the χ^2^-index is sensitive to sample size, we present other fit indices as well. We used the following rules of thumb: CFI > 0.90, RMSEA < 0.08, and SRMR < 0.08 were considered to indicate adequate model fit, whereas CFI > 0.95 and RMSEA < 0.05 were considered to indicate good model fit [57–59]. The internal consistency of the scale was evaluated by Cronbach’s alpha, both for the global factor and for the five lower-level factors.

To use mean scale scores to compare different groups, the scale needs to be measurement invariant with regard to the groups [60–62]. If a scale lacks measurement invariance, observed group differences may be due to differences in the measured constructs, but they may also be due to differences in how the members of the groups interpret the individual items. We therefore evaluated the measurement invariance of the PSMEC scale with regard to gender and time of education (the middle or end of education) by estimating nested CFA models. First, a multigroup CFA model with five factors and no constraints across groups was estimated to establish configural invariance, i.e., to establish that the same number of factors and factor structure (the same pattern of zero and nonzero loadings) fit in both groups. Next, thresholds were constrained to be equal across groups to establish equal distributions for the underlying continuous variables. Finally, the loadings were constrained to be equal across groups to establish scalar invariance, i.e., that group members interpret items similarly, such that mean scores can be compared between the groups. Changes in model fit indices between models are usually evaluated against rules of thumb to evaluate measurement invariance [63, 64]. This is, however, less appropriate when using an estimator for ordinal data [65], and we therefore used the previously mentioned cut-off values for the CFI, RMSEA, and SRMR to evaluate whether the most constrained model fit the data and, if so, to assume scale invariance.

Because some of the items had zero frequencies in the lowest category, these categories were collapsed with their adjacent category for those specific items. In the invariance analysis for gender, categories were collapsed for four items, and in the time in education analysis, categories were collapsed for seven items.

## Results

**Fig. 1:**
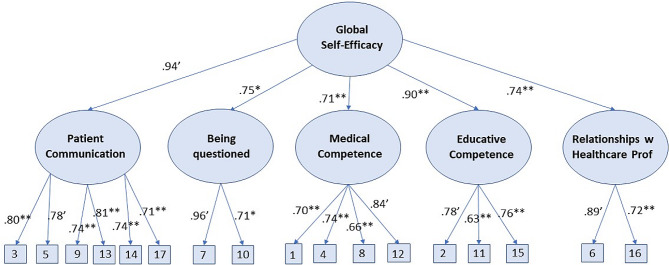
Estimated hierarchical CFA model. Standardized loadings. ‘One unstandardized loading for each factor was fixed to 1. *p <.05, **p <.001

The hierarchical CFA model with one global second-order factor and five first-order factors fit well, χ^2^(114) = 305.828, CFI = 0.989, RMSEA = 0.051 (90%c.i.: 0.044; 0.058), SRMR = 0.055, indicating that the scale can be used to measure the overall construct of self-efficacy as well as the five dimensions of the construct, i.e., the subconstructs. The estimated model is shown in Fig. 1. As shown, “patient communication” and “educative competence” have the highest loadings on the global self-efficacy factor. In addition, items 3 (“I can communicate with ‘difficult’ patients”) and 13 (“I am good at calming patients down”) have the highest loadings on “patient communication”. Item 7 (“I can handle being questioned by patients”) has the highest loading on “being questioned”, item 12 (“I believe in my ability to make sound medical decisions”) has the highest loading on “medical competence”, items 2 (“I can make patients follow advice and recommendations”) and 15 (“I can explain in a way that patients understand”) have the highest loadings on “educative competence”, and item 6 (“I am able to contribute to a positive collaboration within the medical team”) has the highest loading on “relationships with healthcare professionals”.

Chronbach’s α indicated that the global scale showed good internal consistency (α = 0.912). The five dimensions showed acceptable to good internal consistency [66]: “patient communication” (α = 0.870), “being questioned” (α = 0.777), “medical competence” (α = 0.804), “educative competence” (α = 0.727), and “relationships with healthcare professionals” (α = 0.708).

Fit indices from five-factor multigroup CFA analyses are shown in Table 2, with the top of the table showing results for gender as the grouping variable and the bottom of the table showing results with time in education as the grouping variable. As shown, all models fit adequately or well, indicating that the scales measure the same number of dimensions equally for males and females and for students in the middle and at the end of their education. The scales thus show scalar measurement invariance, and mean scores can be compared across groups.

**Table 2:** Fit statistics for measurement invariance evaluations of self-efficacy scale for gender and time in education

Invariance	χ^2^(df)		CFI	RMSEA	SRMR
Gender	(n_1_ = 258, n_2_ = 397)				
Configural	298.154(218)		0.995	0.034(0.023;043)	0.055
Thresholds	464.855(276)		0.989	0.046(0.038;053)	0.055
Thresholds & Loadings	542.729(288)		0.985	0.052(0.045;059)	0.060
Time	(n_1_ = 335, n_2_ = 320)				
Configural	314.566(218)		0.994	0.037(0.027;046)	0.056
Thresholds	500.878(274)		0.986	0.050(0.043;057)	0.057
Thresholds & Loading	550.286(286)		0.984	0.053(0.047;060)	0.060

## Discussion

In this study, we describe the development and validation of a scale measuring physician self-efficacy to manage emotional challenges (PSMEC). The scale was developed to measure five dimensions of the self-efficacy construct: communication with difficult patients and delivering bad news, being questioned and challenged, medical knowledge and competence, educative competence in patient encounters, and ability to establish and maintain relationships with healthcare professionals. CFAs confirmed the two-level factor model with medical students’ physician self-efficacy to manage emotional challenges as the global construct and the five subscales as its dimensions in a sample of Swedish medical students. The scale also demonstrated good internal consistency in measuring medical students’ physician self-efficacy as a global construct, and its five subscales showed acceptable to good internal consistency in measuring the five dimensions. The subscale “educative competence” had only three items, while the subscales “being questioned” and “relationships with healthcare professional” had only two items, which might explain why their alpha values were slightly lower than those of the subscales “patient communication” and “medical competence”.

Measurement invariance analyses supported scalar invariance with regard to gender and time in education. The physician self-efficacy scale can thus be used to evaluate differences in physician self-efficacy both between male and female students and for students in the middle and at the end of their education. Research has found that female students suffer to a larger degree from distress [39, 67], and studies exploring potential gender differences regarding correlations between medical students’ physician self-efficacy beliefs in relation to challenging situations and experiences of distress would be an important contribution to understanding factors influencing student well-being.

The self-efficacy scale reported here was developed to specifically measure medical students’ physician self-efficacy to manage emotional challenges. Therefore, the current inventory complements other instruments, such as the MASS [41] and the Self-efficacy in Medical School inventory [42], which have a comprehensive approach and include all competencies in frameworks such as CanMED and ACGME. According to Bandura, self-efficacy is context dependent, and the level of specificity of a measurement influences the explanatory and predictive value, i.e., domain- and task-specific measurements are preferable for explaining differences and variations in students’ self-efficacy beliefs [36, 48]. The PSMEC scale has been developed to specifically target what students find emotionally challenging, and this scale is therefore useful for investigating students’ beliefs in their ability to handle these kinds of situations. Several studies report on students experiencing emotional challenges during medical education [3, 4, 6, 12, 14] and that they feel inadequate and have self-doubt [4, 7, 26]. Self-efficacy beliefs relate to students’ well-being and resilience [38, 39, 68] and are also important for medical decision-making [37]. We predict that the PSMEC scale will be a useful tool when exploring self-efficacy in future studies investigating medical students’ and junior doctors’ development and/or well-being when dealing with emotionally challenging situations during clinical education and practice. The PSMEC does not assess students’ ability to handle emotionally challenging situations, but rather their belief in their capability to manage. However, given that self-efficacy beliefs are positively correlated to well-being and learning [33, 34, 38, 39], the PSMEC could offer valuable data in research examining well-being and management of stressful situations. Furthermore, the scale can be used to measure the global construct or the five subconstructs (communication, being questioned, medical and educative competence, and relationships with health care teams), depending on the aim of the study.

The PSMEC scale was developed and validated in the context of medical education in Sweden. One strength of the current study, evaluating some of the psychometric properties of the instrument, is that students from all seven medical schools in Sweden participated in answering the questionnaire. The analyses are thus based on data on a national level and are not limited to data from one or a few medical schools. However, limitations of the current study include the high nonresponse rate (72%) and the fact that the sample was from only one country. However, the sample may be considered large enough to avoid convergence problems and biased estimates (see e.g. 67), although the response rate of 28% may have resulted in systematic bias if the respondents differed from the nonrespondents in important ways. The gender distribution (61% female students) corresponds to the gender distribution among medical students enrolled in medical schools in Sweden [50], however respondents and non-respondents may have differed in other ways. Our Swedish sample also limits the generalizability of the findings, however we assume the PSMEC scale, if proven valid in other settings, may be of interest for medical schools outside Sweden. Because the focus of the scale, and that students’ distress is well demonstrated in many other counties [2, 69, 70], the PSMEC could be useful for investigating self-efficacy in managing emotions in other contexts. Thus, future studies should investigate the validity and usefulness of the medical students’ physician self-efficacy to manage emotional challenges scale in other settings and contexts.

Another limitation of the current study is that neither construct validity nor criterion validity were examined. Future studies should, therefore, include the MASS scale and the Self-efficacy in Medical School inventory to test the PSMEC scale’s construct validity and other instruments to measure variables that have been found to be associated with physician self-efficacy (e.g., medical students’ well-being, motivation, learning, and achievement) to test the criterion validity of the PSMEC scale.

We found that the PSMEC scale can be used to compare medical students in the middle and at the end of their education. Our analyses were, however, based on cross-sectional data. Future studies may adopt a longitudinal design to examine test-retest reliability, time measurement invariance, and predictive validity of the scale and to investigate whether there is a change in physician self-efficacy to manage emotional challenges over time.

## Conclusions

Our analyses suggest that the PSMEC scale demonstrated satisfactory psychometric properties with regards to dimensionality, internal consistency, and gender and time of education measurement invariance and that it therefore can be used as a reliable and valid tool for measuring medical students’ physician self-efficacy to manage emotional challenges in work-based education, both as a global construct and as subconstructs: communication with difficult patients and delivering bad news, being questioned and challenged, medical knowledge and competence, educative competence in patient encounters, and ability to establish and maintain relationships with healthcare professionals. In addition, this study supports the use of the PSMEC scale when comparing global or subconstruct means for male and female medical students and for students at the middle and at the end of their undergraduate medical education.

### Electronic supplementary material

Below is the link to the electronic supplementary material.

Supplementary Material 1

## Data Availability

Data will be available upon request from the corresponding author.

## Abbreviations

ACGME: Accreditation Council for Graduate Medical Education
CanMEDs: Physician Competency Framework, Canada
PSMEC: Physician Self-Efficacy to Manage Emotional Challenges
CFA: Confirmatory Factor Analysis
COVID-19: SARS-Coronavirus-2 discovered in 2019
CFI: Comparative Fit Index
RMSEA: Root Mean Square Error of Approximation
SRMR: Standardized Root Mean squared Residual
