# Supplementary material for: Development and validation of the physician self-efficacy to manage emotional challenges Scale (PSMEC)
Source: BMC Med Educ. 2024 Mar 4;24:228. doi: 10.1186/s12909-024-05220-9 (PMC10913217; doi:10.1186/s12909-024-05220-9)
Supplement: Supplementary file 1 — Supplementary Material 1 [file 12909_2024_5220_MOESM1_ESM.pdf]

Appendix 1. The items of the Physician Self-efficacy to Manage Emotional Challenges Scale (PSMEC)

Items
1. I feel confident that I have sufficient knowledge
2. I can make patients follow advice and recommendations
3. I can communicate with difficult patients
4. I am good at the practical skills that are necessary in my role as a physician
5. I can handle difficult questions from patients
6. I am able to contribute to a positive collaboration in the healthcare team
7. I can handle being questioned by patients
8. I feel confident in how to handle my knowledge gaps
9. I am confident in my ability to meet a patient/relative who expresses strong emotions
10. I can handle being questioned by healthcare professionals
11. I am good at judging whether the patient has understood my information
12. I believe in my ability to make sound medical decisions
13. I am good at calming patients down
14. I am good at forming good relations to patients
15. I can explain in a way that patients understand
16. I am good at establishing good relations to the healthcare professionals
17. I am good at delivering bad news to the patient

The response scale for each item is a six-point Likert-type scale from 1 = strongly disagree to 6 = strongly agree.
